# Cortical Reorganization after Hand Immobilization: The beta qEEG Spectral Coherence Evidences

**DOI:** 10.1371/journal.pone.0079912

**Published:** 2013-11-22

**Authors:** Marina Fortuna, Silmar Teixeira, Sérgio Machado, Bruna Velasques, Juliana Bittencourt, Caroline Peressutti, Henning Budde, Mauricio Cagy, Antonio E. Nardi, Roberto Piedade, Pedro Ribeiro, Oscar Arias-Carrión

**Affiliations:** 1 Brain Mapping and Sensory Motor Integration, Institute of Psychiatry of Federal University of Rio de Janeiro (IPUB/UFRJ), Rio de Janeiro, Brazil; 2 School of Physical Education, Bioscience Department (EEFD/UFRJ), Rio de Janeiro, Brazil; 3 Division of Epidemiology and Biostatistic, Institute of Health Community, Federal Fluminense University (UFF), Rio de Janeiro, Brazil; 4 Institute of Applied Neuroscience (INA), Rio de Janeiro, Brazil; 5 Laboratory of Physical Therapy, Veiga de Almeida University, Rio de Janeiro, Brazil; 6 Physical Therapy Department, Piquet Carneiro Policlinic, State University of Rio de Janeiro (UERJ), Rio de Janeiro, Brazil; 7 Panic and Respiration, Institute of Psychiatry of Federal University of Rio de Janeiro, Rio de Janeiro, Brazil; 8 National Institute for Translational Medicine (INCT-TM), Rio de Janeiro, Brazil; 9 Quiropraxia Program of the Faculty of Health Sciences, Central University, Santiago, Chile; 10 Physical Activity Neuroscience, Physical Activity Sciences Postgraduate Program – Salgado de Oliveira University, Niterói, Brazil; 11 Institute of Phylosophy, Federal University of Uberlândia (IFILO/UFU), Rio de Janeiro, Brazil; 12 Medical School Hamburg, University of applied science and Medical University. Hamburg, Germany; 13 Movement Disorders and Transcranial Magnetic Stimulation Unit, Hospital General Dr. Manuel Gea González, México D.F., México; 14 Neurology department, Hospital General Ajusco Medio, México D.F., México; Universitätsklinikum Carl Gustav Carus an der Technischen Universität Dresden, Germany

## Abstract

There is increasing evidence that hand immobilization is associated with various changes in the brain. Indeed, beta band coherence is strongly related to motor act and sensitive stimuli. In this study we investigate the electrophysiological and cortical changes that occur when subjects are submitted to hand immobilization. We hypothesized that beta coherence oscillations act as a mechanism underlying inter- and intra-hemispheric changes. As a methodology for our study fifteen healthy individuals between the ages of 20 and 30 years were subjected to a right index finger task before and after hand immobilization while their brain activity pattern was recorded using quantitative electroencephalography. This analysis revealed that hand immobilization caused changes in frontal, central and parietal areas of the brain. The main findings showed a lower beta-2 band in frontal regions and greater cortical activity in central and parietal areas. In summary, the coherence increased in the frontal, central and parietal cortex, due to hand immobilization and it adjusted the brains functioning, which had been disrupted by the procedure. Moreover, the brain adaptation upon hand immobilization of the subjects involved inter- and intra-hemispheric changes.

## Introduction

During their lifetime, human beings are highly likely to suffer accidents that can provoke hand immobilization (HI), which may temporarily or permanently impair the hand movements, and consequently, may induce changes in the cortical organization [1], [2], [3], [4], [5], [6], [7]. In particular, changes appear in the primary somatosensory cortex and primary motor areas (M1) in response to alterations of the hand conditions [8], [9]. In this study we investigate the cortical changes occurring after HI. For this purpose, we applied quantitative electroencephalography (qEEG), which has been proven to be a useful tool to examine cortical changes after limb immobilization [10], [11]. Specifically, qEEG can be employed to observe electro-cortical alterations produced by HI of the subject´s right hand [11], [12]., We used coherence measures on beta band (β) (12 to 30Hz) which has been related to pre-motor and motor cortical regions during movement, attention tasks and cognitive functions in general [13], [14].

In addition, coherence measures estimates the coupling between two cortical areas that is suitable to observe changes during a sensorimotor task [15], [16]. A coherence decrease appears to act as a neural tracer of implicit memory (i.e., motor procedures) [16]. For this reason, in our experiment we used coherence to examine beta bands on the prefrontal and premotor scalp areas that are associated with motivation, planning and motor programming (F3-FZ/F4-FZ/F3-F4). In addition, we observed electrodes derivation which represent in prefrontal and premotor areas (F3/F4, F3/FZ, F4/FZ and F7/F8), motor cortex (C3/C4, C3/CZ and C4/CZ), parietal areas (P3/P4, P3/PZ and P4/PZ) and finally, secondary motor areas (T3/F7, T4/F8, T3/T4 and T5/T6). Here, we hypothesized that greater coupling occurs within inter- and intra-hemispheric cortical areas due to a new motor planning and sensorimotor integration to execute motor acts after hand immobilization.

## Results

**Figure 1:**
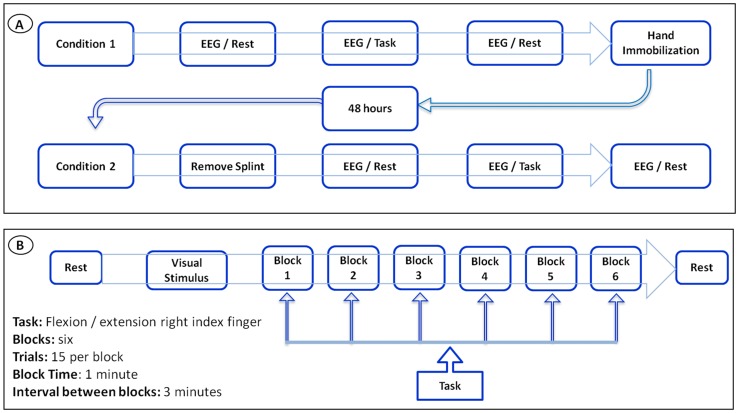
Experimental Setup. a) Complete experimental design; b) Condition of the experimental design.

**Figure 2:**
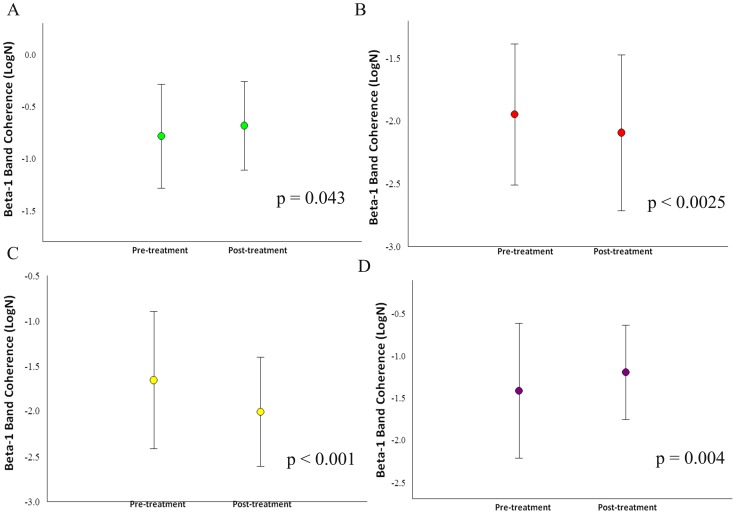
Treatment's main effects observed in the frontal and central electrodes by mean and SD for beta-1 coherence. a) Electrode F4/FZ (p = 0.043); b) Electrodes F7/F8 (p = 0.025); c) Electrodes F7/FZ (p<0.001); d) electrodes C4/CZ (p = 0.004).

**Figure 3:**
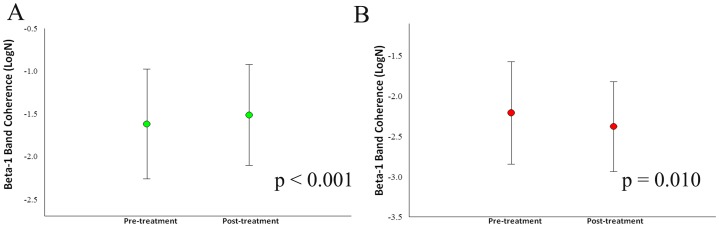
Treatment's main effects observed in the parietal and temporal electrodes by mean and SD for beta-1 coherence. a) Electrode P4/PZ (p<0.001). b) Electrodes T5/T6 (p = 0.010).

**Figure 4:**
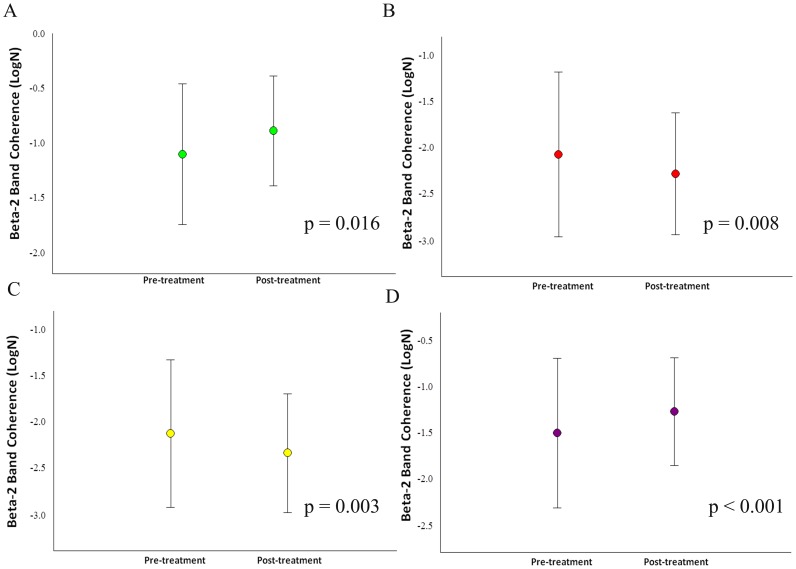
Treatment's main effects observed in the frontal and central electrodes by mean and SD for beta-2 coherence. a) Electrodes F4/FZ (p<0.001); b) Electrodes F7/FZ (p = 0.016); c) Electrodes C3/C4 (p = 0.008); d) Electrodes C4/CZ (p = 0.003).

**Figure 5:**
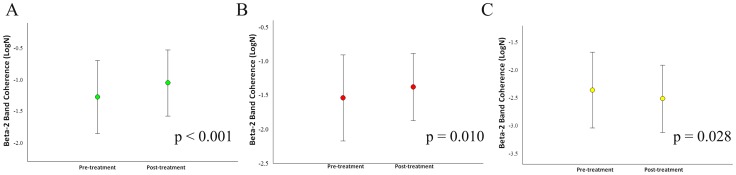
Treatment's main effects observed in the parietal and temporal electrodes by mean and SD for beta-2 coherence. a) Electrodes P3/PZ (p<0.001); b) Electrodes P4/PZ (p = 0.010); c) Electrodes T5/T6 (p = 0.028).

**Figure 6:**
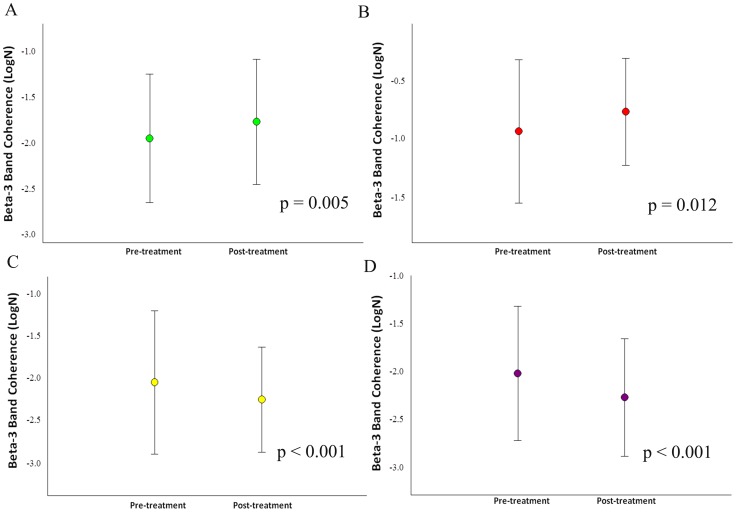
Treatment's main effects observed in the frontal electrodes by mean and SD for beta-3 coherence. a) Electrodes F3/F4 (p = 0.018); b) Electrodes F3/FZ (p = 0.005); c) Electrodes F7/FZ (p = 0.012); d) Electrodes C3/C4 (p<0.001).

**Figure 7:**
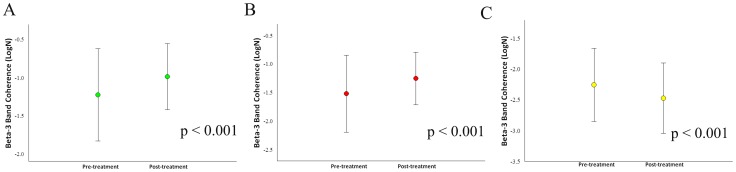
Treatment's main effects observed in the parietal electrodes by mean and SD for beta-3 coherence. a) Electrodes P3/PZ (p<0.0001); b) Electrodes P4/PZ (p<0.0001); c) Electrodes T5/T6 (p<0.001).

Our sample was composed of 6 cell-blocks, for a total of 12 cells for each participant. Thus, there are 90 cells for each condition and 180 cells per visit to the lab. For the whole design, we used 330 cells. The missing values (i.e., 30) were accounted for. Thus, our sample size was composed of 6 cells for each block, totalizing 12 cells for each participant (Figure 1A, B). Thus, we have a total of 90 cells for each condition and 180 cells per visit to the lab. For the whole design we used 330 cells. The missing values were accounted (i.e., 30). Thus, our results from the ANOVA did not show any interaction or moment main effect for index finger movement. However, we found treatment main effect (i.e., pre-immobilization vs. post-immobilization) for the derivations at beta-1 (β1): F4/FZ [*F*(1,330) = 4.144; p-value  = 0.043] (Figure 2A); F7/F8 [*F*(1,330) = 5.070 = p-value  = 0.025] (Figure 2B); F7/FZ [*F*(1,330)  = 21.963; p-value <0.001] (Figure 2C); C4/CZ [*F*(1,330)  = 8.332; p-value  = 0.004] (Figure 2D); P4/PZ [F(1,330)  = 10.860; p-value <0.001] (Figure 3A); and T5/T6 [*F*(1,330)  = 6.771; p-value  = 0.010] (Figure 3B). For the derivations at beta-2 (β2): F4/FZ [*F*(1,330)  = 19.283; p-value <0.001] (Figure 4A); F7/FZ [*F*(3,330)  = 5.899; p-value  = 0.016] (Figure 4B); C3/C4 [*F*(1,330)  = 7.135; p-value  = 0.008] (Figure 4C); C4/CZ [*F*(1,330)  = 9.054; p-value  = 0.003] (Figure 4D); P3/PZ [*F*(1,330)  = 13.687; p-value <0.001] (Figure 5A); P4/PZ [*F*(1,330)  = 6.654; p-value  = 0.010] (Figure 5B) and T5/T6 [*F*(1,330)  = 4.843; p-value  = 0.028] (Figure 5C). For the derivations at beta (β3): F3/F4 [*F*(1,330)  = 5.642; p-value  = 0.018] (Figure 6A); F3/FZ [*F*(1,330)  = 7.944; p-value  = 0.005] (Figure 6B); F7/FZ [*F*(1.330)  = 6.437; p-value  = 0.012] (Figure 6C); C3/C4 [*F*(1.330)  = 12.144; p-value <0.001] (Figure 6D); P3/PZ [*F*(1.330)  = 17.208; p-value <0.001] (Figure 7A); P4/PZ [*F*(1.330)  = 17.208; p-value <0.001] (Figure 7B); and T5/T6 [*F*(1.330)  = 11.545; p-value <0.001] (Figure 7C). (See all results in table I).

**Table 1:** Statistical results for treatment's main effect (pre-immobilization vs. post-immobilization).

Paired Electrodes	Two way ANOVA at β1 sub-band	Two way ANOVA at β2 sub-band	Two way ANOVA at β3 sub-band
**F3/F4**	*F*(1,330) = 0.828; *p* = 0.363	*F*(1,330) = 2.517; *p* = 0.114	*F*(1,330) = 5.642; *p* = **0.018**
**F3/FZ**	*F*(1,330) = 2.116; *p* = 0.147	*F*(1,330) = 2.480; *p* = 0.116	*F*(1,330) = 7.944; *p* = **0.005**
**F4/FZ**	*F*(1,330) = 4.144; *p* = **0.043**	*F*(1,330) = 19.283; *p*<**0.001**	*F*(1,330) = 3.774; *p* = 0.053
**F7/F8**	*F*(1,330) = 5.070; *p* = **0.025**	*F*(1,330) = 0.160; *p* = 0.690	*F*(1,330) = 0.023; *p* = 0.881
**F7/FZ**	*F*(1,330) = 21.963; *p*<**0.001**	*F*(3,330) = 5.899; *p* = **0.016**	*F*(1,330) = 6.437; *p* = **0.012**
**F8/FZ**	*F*(1,330) = 0.414; *p* = 0.520	*F*(1,330) = 1.090; *p* = 0.297	*F*(1,330) = 0.085; *p* = 0.770
**C3/C4**	*F*(1,330) = 2.584; *p* = 0.109	*F*(1,330) = 7.135; *p* = **0.008**	*F*(1,330) = 12.144; *p*<**0.001**
**C3/CZ**	*F*(1,330) = 0.341; *p* = 0.560	*F*(1,330) = 0,147; *p* = 0.702	*F*(1,330) = 2.116; *p* = 0.147
**C4/CZ**	*F*(1,330) = 8.332; *p* = **0.004**	*F*(1,330) = 9.054; *p* = **0.003**	*F*(1,330) = 2.305; *p* = 0.130
**P3/P4**	*F*(1,330) = 0.841; *p* = 0.360	*F*(1,330) = 0.855; *p* = 0.356	*F*(1,330) = 0.195; *p* = 0.659
**P3/PZ**	*F*(1,330) = 7.643; *p* = **0.006**	*F*(1,330) = 13.687; *p*<**0.001**	*F*(1,330) = 17.208; *p*<**0.001**
**P4/PZ**	F(1,330) = 10.860; *p*<**0.001**	*F*(1,330) = 6.654; *p* = **0.010**	F(1,330) = 17.705; *p*<**0.001**
**T3/T4**	*F*(1,330) = 0.552; *p* = 0.458	*F*(1,330) = 3.026; *p* = 0.083	F(1,330) = 1.995; *p* = 0.159
**T5/T6**	*F*(1,330) = 6.771; *p* = **0.010**	*F*(1,330) = 4.843; *p* = **0.028**	*F*(1,330) = 11.545; *p*<**0,001**

The significance of bold values is *p*<0.05.

## Discussion

The objective of this study is to shed light on the cortical coupling changes that occur when individuals are submitted to hand restriction. In particular, we will discuss the effects after 48 hours of HI on the frontal, central, parietal and temporal regions. Based on previous electrophysiological findings, we hypothesized that beta band coherence oscillations act as main neural mechanism underlying information processing, which changes after hand immobilization. We expected that a greater coupling occurred within inter- and intra-hemispheric areas due to new motor planning and sensorimotor integration to execute motor acts after hand immobilization. Moreover, we anticipated that changeover occurred in the beta band coherence, when comparing its level before and after hand immobilization.

Our results at beta-2 for F3/F4, F3/FZ, F7/FZ, F3/F4 and F7-F8 derivations were not significant. We speculated that the cortical changes caused by HI occurred in superficial cortical layers and, thereby, the β2 band was not as expressive [28]. On the other hand, we reasoned that β3 band better represented the coherence for F3/F4 and F3/FZ derivations. The β3 band showed that the inter- and intra-hemispheric coherence is needed to plan [29] the motor act. Moreover, the inter-hemispheric relationship of the sensory information through the corpus callosum [30] may be one factor that provoked coherence increase in F3/F4 derivations. Furthermore, we found coherence increase in F7/F8 derivations from β1 band and F7/FZ from β1 and β3 bands. We concluded that the secondary motor area had functional autonomy due to the fact that HI did not cause new task demands. Thus, the F7/F8 and F7/FZ derivations did not need more coupling to prepare and to select the movement during the task after HI [31]. Specifically, the β1 band has been related with complex associative functions [8]. Furthermore, we observed in F7/F8 and F7/FZ derivations that coherence decreased; these derivations are related to associative function and are thereby responsible for the pre-programing of sequential actions (i.e., flexion and extension of the index finger) [32]. Moreover, the subjects waited for the visual stimuli on the computer screen to execute the task; this may have been a factor that produced coherence decrease for F7/F8 and F7/FZ derivations because these regions inhibit cognitive neural processes that are not needed during the task [33]. i.e., our task did not recover motor planning memory. On the other hand, coherence increased for F3/F4 and F3/FZ at β3 band; we associated this with studies that related more coupling in F3/F4 and F3/FZ derivations in tasks that needed automatic movement [34]. Furthermore, our findings about the coherence increase of these electrode derivations contrasted with the study by Wada et al. [35] that showed low inter-hemispheric coherence at β band when subjects are under alert condition.

Experiments have shown that cortical representation changes in motor areas when subjects execute a motor act [36]. Specifically, the M1 has been related to high reorganization capacity when subjects execute simple movements [37]. Other studies were also found to be in agreement with our results, which showed coherence decreased for C3/C4 derivations at β2 and β3 bands in M1 after HI [38], [39]. This probably occurred because the finger movement may have changed the pattern excitability in M1 in healthy subjects when training new abilities [40], [41]. Therefore, the HI induced changes at these derivations (i.e., C3/C4) where coherence decrease may be an indication of specialization of the M1 [16]. Furthermore, we related the coherence increase for C4/CZ derivations at β1 and β2 bands with the results for P4/PZ derivations, considering the adjustments which occurred in the parietal and fronto-parietal [44]; consequently, the coupling was higher for C4/CZ derivations. Then, we suggested that HI promoted such cortical changes so that, when subjects executed the task after HI, the ipsilateral hemisphere needed to readjust cortical functions. Moreover, when the subjects had their right hand immobilized, the contralateral hand maintained normal movement; this probably increased the coupling for C4/CZ derivations. In addition, our findings from central derivations may be associated with the sequential movement coordination [42], and future motor action organization and planning [43].

The cerebral mapping has demonstrated strong evidence of the parietal cortex involvement, mainly in planning and movement control, when subjects were exposed to the motor task [31]. Furthermore, our results for P3/PZ and P4/PZ derivations at β1, β2 and β3 bands showed increased coherence. This finding demonstrated that only intra-hemispheric coherence occurred in in the parietal cortex of both hemispheres. The increased coupling in the contralateral parietal cortex (i.e., P3/PZ derivations) may be due to movement adjustment after HI [44] and many reflect the role of the parietal cortex in motor performance [45]. Moreover, when the subjects executed the index finger movement, the parietal cortex codified its position space and temporal information of the index finger movement [46], [47]. In addition to this, coherence increase in the ipsilateral parietal cortex (i.e., P4/PZ derivations) may result from the new sensorial organization after HI [48]. Thereby, the ipsilateral parietal cortex adjusted sensory integration and sent it to the frontal regions [49]. Thus, our findings for P3/PZ and P4/PZ derivations were not consistent with the study by Sainburg [50] who showed that, when subjects executed the task, new information was created and the contralateral activity increased while the ipsilateral activity decreased.

Finally, we found that coherence decreased for T5/T6 derivations at β1, β2 and β3 bands. We explored the hypothesis that HI did not generate cortical changes from the motor memory management. This result is in agreement with studies, which showed that the temporal cortex activity decreased when subjects were executing a task that did not need memory management [51], while in the parietal cortex the neural activity increased [52]. Therefore, our findings are in agreement with the idea that when individuals execute a task which implicates working memory, the cerebral cortex needs to activate other cortical circuits [51]. Our data at T5/T6 derivation may indicate that neural implicit memory did not occur after HI [16].

Thus, it was demonstrated that HI introduced changes in the temporal cortex. The pre-frontal, M1 and parietal cortex, in order to facilitate cortical circuits and to execute necessary cortical adjustments, so that the motor response (i.e., index finger movement) in generated [23].

This study aimed at shedding light on the hand immobilization effects on the beta band coherence. Particularly, we investigated the differences among paired electrodes in the frontal, central and parietal cortices during an index finger flexion and extension task. Based on previous electrophysiological findings, we hypothesized that beta band coherence oscillations would show greater inter- and intra-hemispheric activation. However, we did not find highter inter- and intra-hemispheric activity for all derivations. Nonetheless, our findings provided the possibility to analyze the fact that β2 band was not more active in frontal regions after HI. The coherence predominance was at β1 and β3 bands. Moreover, HI caused changes in the coherence of electrode derivations related with planning, sensory integration and motor act. These alterations occurred more for the inter-hemispheric derivations. The coherence increased especially in the parietal cortex in order to adjust the information and send it to the pre-motor areas, so that the subjects could execute the task. Thus, our findings suggest a disrupted connection of the brain functioning after HI i.e., a deregulation occurred in the cortical mechanisms. This reinforced our hypothesis that beta band coherence provides information about changes in cortical areas when subjects are submitted to HI.

## Methods

### Sample

The sample was composed of 15 healthy individuals: 04 men and 11 women, between the ages of 20 and 30 (average age 24±1.2 years). The individuals were chosen randomly and the recruitment of the volunteers was accomplished thanks to research announcements posted in different universities of Rio de Janeiro State. As inclusion criteria, the subjects needed to be right handed, have no mental or physical illness (previous anamnese) and not use any psychoactive or psychotropic substances during the whole time of the study. We applied a detailed questionnaire in order to exclude those individuals who could compromise our results. Due to hand laterality we utilized the Edinburgh inventory [17] to indentify the predominance of the participants (right handed vs. left-handed). Consequently, the left-handed individuals were excluded from the experiment. We instructed the subjects to not consume tobacco, coffee or alcohol 10 hours prior to the test. The participants received written information about the study procedures and we solicited their signature on the consent form. This study was approved by the ethics committee of Veiga de Almeida University through number 149.817 conform laid down in the 1964 Declaration of Helsinki.

### Experimental procedure

A room was prepared with acoustic and electrical isolation. During the EEG signal acquisition, the lights were dimmed. The subjects sat in a chair with armrest in order to minimize muscle artifact during EEG signal acquisition. In front of the subjects a 15-inch monitor was placed on a table in front of the subjects. The monitor was turned on only when the subjects executed the task (i.e., flexion and extension of the index finger). Initially, the EEG signal acquisition lasted for 2 minutes (rest) with the monitor in the off position, facing the subjects. Then, we coupled a sensor (accelerometer) to measure acceleration on the right index finger; during the visual feedback, the subjects executed the task. The accelerometer was connected to the EEG through an additional channel (i.e., channel 21). When the subjects performed the movement, the accelerometer provided a signal for the EEG. The individuals were instructed to perform the index finger flexion and extension when visual feedback was generated by a random image on the monitor. The subjects executed the task in 6 blocks of 15 trials each. In order to avoid muscle fatigue, they rested for 3 minutes between each block. After completing the task, the monitor was turned off and the subjects were submitted again to EEG during 2 minutes (rest). After EEG recording, we applied a sprint on the subjects' right hand and they kept it on for 48 hours. After this period they returned to the laboratory to remove the sprint and they were again submitted again to the task using the same procedures applied prior to hand immobilization (Figure 1).

### Electroencephalography

The International 10/20 system for electrodes was used together with a 20-channel Braintech-3000 EEG system (EMSA-Medical Instruments, Brazil). The 20 electrodes were arranged in a nylon cap (ElectroCap Inc., Fairfax, VA, USA), yielding mono-pole derivations to linked earlobes, which were reference points. In addition, two 9-mm diameter electrodes were attached above and on the external corner of the right eye, in a bipolar electrode montage, to monitor artifacts on eye-movements through Electrooculagraphy (EOG). Impedance of EEG and EOG electrodes was kept under 5–10 KΩ. The data acquired had total amplitude of less than 100 µV. The EEG signal was amplified with a gain of 22,000, analogically filtered between 0.01 Hz (high-pass) and 100 Hz (low-pass), and sampled at 240 Hz. The software Data Acquisition (Delphi 5.0), developed at the Brain Mapping and Sensorimotor Integration Laboratory was employed to filter the raw data: notch (60 Hz), high-pass of (0.3 Hz) and low-pass (100 Hz).

### Accelerometer

In order to obtain signals from the accelerometer we used the model MMA7340 of Freescale semiconductors. This system is formed by microelectronics mechanisms (MEMS), which explore the mechanical proprieties of silicone to create movable structures and to detect distinct movement directions [18], [19], [20]. The capture of movements was conducted in actual time system with the interaction of EEG software signal acquisition. As the movement was performed, the accelerometer showed a curve with acceleration variability providing information about velocity and time.

### Data processing

A visual inspection and independent component analysis (ICA) is an information maximization algorithm to blind the EEG signals related to the artifacts [21]. It was applied to quantify reference-free data, and to identify and remove any remaining artifacts, i.e., eye blinks and ocular movements, produced by the task. Data from individual electrodes exhibiting loss of contact with the scalp or high impedances (>10 kΩ) were discarded, and data from single-trial epochs exhibiting excessive movement artifacts (±100 µV) were also deleted. ICA was then applied to identify and remove any artifacts after the initial visual inspection. Independent components resembling eye-blink or muscle artifacts were removed and the remaining components were then projected back onto the electrode data by multiplying them by the inverse matrix of the spatial filter coefficients derived from ICA using established procedures. The ICA-filtered data were then reinspected for residual artifacts using the same rejection criteria described above. Then, a classic estimator was applied for the power spectral density, or directly from the square modulus of the Fourier Transform, performed by MATLAB (Matworks, Inc.). Quantitative EEG parameters were reduced to 4s-periods (the selected epoch started 2s before and ended 2s after the trigger). For this analysis we excluded the channel 21 created for the accelerometer, in order to avoid artifacts.

### Spatial electrode localization

We selected electrodes in prefrontal and premotor areas (F3/F4, F3/FZ, F4/FZ and F7/F8) due to their functional relationship with planning, stored motor programming and motivations [22]. Other electrodes were selected due to their relationship with the motor cortex (C3/C4, C3/CZ and C4/CZ), [23]. Additionally, electrodes which represent parietal areas (P3/P4, P3/PZ and P4/PZ) were chosen due to their functional relationship with sensorimotor integration [12], [24]. Finally, the electrodes representing temporal areas (T3/F7, T4/F8, T3/T4 and T5/T6) were chosen due to their relationship with motor memory management [25].

### Statistical analysis

The statistical design allowed the examination of functional cortical action before and after HI. We selected areas in each hemisphere, with respective regions related to sensory, motor execution, and integrative or associative functions. We standardized and normalized data into values of absolute power using homocedasticity in a natural logarithmic test (LogN) in order to approximate values to a normal distribution [26], [27]. After this, we used the Kolmogorov-Smirnov test, which showed a normal distribution after changing into LogN. In addition to this, an ANOVA was conducted for the same groups of electrodes for two factors: treatment (before and after HI), and moment (trigger before and after index finger movement). The whole analysis was corrected by multiple comparisons using the *post hoc* test with Bonferroni's correction procedure. According to this test, the significance levels were set at P≤0.05. All analyses were conducted using the SPSS for Windows version 18.0 (SPSS Inc., Chicago, Il, USA).
